# A direct comparison of the measurement properties of the PROMIS-16 and EQ-5D-5L in the U.S. general population

**DOI:** 10.1007/s11136-026-04242-8

**Published:** 2026-05-03

**Authors:** Minh Pham, Benjamin M. Craig, Tessa Peasgood, Fanni Rencz

**Affiliations:** 1 Department of Economics, College of Arts and Sciences, University of South Florida, 4202 E Fowler Ave, Tampa, FL 33620 USA; 2 School of Medicine and Population Health, University of Sheffield, Sheffield, UK; 3 Department of Health Policy, Corvinus University of Budapest, Budapest, Hungary

**Keywords:** PROMIS, EQ-5D-5L, Health-related quality of life, Comparable outcome

## Abstract

**Objectives:**

Introduced in 2024, the PROMIS®-16 is a health-related quality of life (HRQoL) instrument that measures eight dimensions: physical function, ability to participate in social roles and activities, anxiety, depression, sleep disturbance, pain interference, cognitive function, and fatigue. Given the potential overlap in dimensions, this study compares the measurement and psychometric properties of the PROMIS-16 and EQ-5D-5L.

**Methods:**

In 2024, an online cross-sectional survey was conducted with a nationally representative sample of U.S. adults aged 18 years or older (N = 2577), including the EQ-5D-5L and PROMIS-16 as stand-alone instruments. We compared the measurement properties of the PROMIS-16 and the EQ-5D-5L, namely ceiling and floor percentages, informativity (Shannon’s indices), and convergent/divergent validity. Furthermore, we conducted an exploratory factor analysis to identify their combined structure.

**Results:**

Within construct-overlapping items, the PROMIS-16 items exhibited greater dispersion, including lower ceiling percentages and higher informativity, compared to their EQ-5D-5L counterparts. Good convergent validity between PROMIS-16 and EQ-5D-5L items was observed for physical functioning (mobility; r = 0.85) and anxiety/depression (r = 0.76–0.79). EFA captured six distinct constructs: Physical Function, Social Roles & Activities, Anxiety / Depression, Pain Interference, Cognitive Function, and Sleep / Fatigue. Possibly due to reverse coding, the two Cognitive Function items of PROMIS-16 showed inconsistent performance, with weak correlations and limited alignment with other constructs.

**Conclusions:**

The PROMIS-16 items demonstrated greater conceptual coverage and informativity than the EQ-5D-5L, but issues with inconsistent framing and potential response biases remained. On the other hand, the EQ-5D-5L demonstrated stronger concordance with respondents’ global health assessments compared to the PROMIS-16.

**Supplementary Information:**

The online version contains supplementary material available at 10.1007/s11136-026-04242-8.

## Background

In recent years, the use of patient-reported outcome measures (PROMs) to measure health-related quality of life (HRQoL) has grown significantly with the advancement of modern healthcare [1–3]. With their accessibility and broad applicability, generic PROMs are accessible tools that can be applied broadly across healthcare settings to evaluate patient quality of life. By providing detailed insights into HRQoL dimensions, they inform treatment and management decisions for both healthcare providers and patients [4, 5]. Furthermore, the collection of PROMs data serves as critical resources for health systems and policymakers, informing decisions about resource allocation, treatment guidelines, and health policy development. In both the United States and internationally, HRQoL is frequently assessed using instruments developed by the Patient-Reported Outcomes Measurement Information System (PROMIS®) Health Organization (promishealth.org) and the EuroQol Group (euroqol.org) [6–11].

In February 2024, Edelen and colleagues introduced a new generic HRQoL instrument derived from four sources of items (PROMIS-29+2, PROPr-14, UPMC16, SIGNAL): the PROMIS-16 profile [12, 13]. Developed to address limitations of earlier PROMIS Profile measures, the PROMIS-16 was designed as a short, clinically practical alternative to instruments like the PROMIS-29 v2.0 and PROMIS Global-10, with potential use in research and clinical care [12, 13]. While the PROMIS-29 v 2.0 was the shortest profile available at the time, its length could still pose challenges for patients and healthcare providers [12, 14]. Meanwhile, the PROMIS Global-10 is not a profile instrument and lacks dimension-specific scores needed for routine clinical use [12, 15]. The PROMIS-16 includes eight dimensions, each measured by two five-level items: physical function (PF), ability to participate in social roles and activities (SOC), pain interference (PI), anxiety (ANX), depression (DEP), sleep disturbance (SLP), cognitive function (COG), and fatigue (FTG) [12].

The performance of items may differ between development-purpose item-testing surveys (i.e., when completed alongside multiple similar items) and surveys with stand-alone instruments due to measurement context dependencies. To date, the PROMIS-16 items have only been administered alongside other PROMIS items in item-testing surveys, and no prior survey has used the PROMIS-16 as a stand-alone instrument. Analyses from prior studies suggest comparable dimension-specific T-scores to longer PROMIS profiles; however, because PROMIS-16 responses were taken directly from PROMIS-29 administrations, such similarity is largely expected by construction [12, 16–19]. As such, further research is needed to validate PROMIS-16 as a stand-alone instrument and compare its performance with other instruments, such as the EQ-5D-5L.

The EQ-5D-5L is a widely used preference-based measure of HRQoL, comprising a parsimonious descriptive system with five dimension-specific items—mobility, self-care, usual activities, pain/discomfort, and anxiety/depression—each rated on five levels of severity, along with a visual analogue scale (EQ VAS) that captures self-rated health on a scale from 0 (the worst health you can imagine) to 100 (the best health you can imagine) [8, 9, 20–23]. PROMIS-16 exhibits partial conceptual overlap with the EQ-5D-5L, as both instruments capture key constructs of HRQoL such as physical functioning (mobility), anxiety, and depression. At the same time, important differences remain: whereas all EQ-5D-5L dimensions use a ‘today’ recall period with five categorical severity levels, PROMIS-16 dimensions (except PF and SOC) are assessed with a 7-day recall period with frequency or difficulty response scales [12, 23].

These differences make it informative to examine how distinct approaches to measuring HRQoL behave when administered in the same population. The EQ-5D-5L was selected as the comparator for PROMIS-16 because it is one of the most widely used generic HRQoL measures in population health research, clinical studies, and health economic evaluation, providing a well-established reference for interpreting measurement properties [8, 9, 20, 21]. Although longer PROMIS Profile measures have been compared with the EQ-5D in prior research, it remains unclear whether such findings extend to ultra-short instruments like PROMIS-16 [24]. In the absence of a closer non-PROMIS alternative that is short, widely validated, and relevant for population-level assessment and economic applications, the EQ-5D-5L represents the most appropriate external comparator.

Beyond descriptive performance, differences between PROMIS-16 and EQ-5D-5L may also affect how dimensions relate to respondents’ overall perceptions of health. As such, examining correlations with the EQ VAS helps contextualize how each instrument related to overall self-rated health and supports validity testing. In addition, PROMIS-16 represents each dimension with two items, whereas the EQ-5D-5L represents each dimension with a single item, which affects how item responses contribute to shared variance at the instrument level. Examining the latent structure of each instrument therefore provides essential context for interpreting observed distributional patterns and relationships with self-rated health.

This study evaluated the measurement properties of PROMIS-16 with the EQ-5D-5L. Specifically, we aimed to: (1) compare PROMIS-16 with EQ-5D-5L in terms of distributional characteristics, ceiling and floor percentages, informativity, and validity (including construct, convergent, and divergent validity); and (2) assess the linear relationship between respondent profiles—modeled separately for PROMIS-16 and EQ-5D-5L items and self-reported health status as measured by the EQ VAS; and (3) explore the underlying construct of the PROMIS-16 and EQ-5D-5L instruments by conducting exploratory factor analysis (EFA).

## Methods

### Data

The independent review board (IRB; Advarra, Pro00072276; 27 June 2023) reviewed the protocol for the original study and determined that it is exempt from IRB oversight based on the U.S. Department of Health and Human Services regulations found at 45 CFR 46.104(d)(2) [25–27]. Between March 18th and April 6th, 2024, we conducted the second wave of the online survey and recruited 4989 adults aged 18 or older from the U.S. general population (Dynata). Respondents were recruited to fill 18 demographic quotas based on gender (male, female and other), age (18–34, 35–54, and 55 and older), and race/ethnicity (Hispanic, non-Hispanic Black, and non-Hispanic other). Instead of excluding interested participants who belong to filled quotas, the recruitment strategy targeted potential participants who belong to unfilled quotas until the quotas were achieved. Inclusion criteria required participants to reside in one of the 50 U.S. states or Washington, D.C. and meet one of 18 demographic quotas. Exclusion criteria (applied after the screener) included failing a comprehension quiz, incomplete surveys, or completing the survey in under 13 min.

Among the respondents who started the survey, 248 (5%) dropped out before being assigned to a study arm, and 1742 (35%) were excluded due to failing to pass a quiz assessing their understanding of being in a coma after three attempts. Among those who passed the screening and background components, 422 (8%) voluntarily dropped out during the preference elicitation tasks or follow-up questions, leaving an analytical sample of 2577 (52%) respondents who completed the survey fully. This analytical sample includes respondents from all 50 U.S. states and Washington, D.C., and their characteristics align with the 2021 American Community Survey (ACS) 1-year estimate for US adults (age 18 or older) except for certain socioeconomic characteristics (never married, some college, and $150,000 or more annual income; see Appendix 1).

### Survey instrument

The survey instrument consisted of five components and was administered fully electronically [25–27]. The consent and screener sections included six questions comprising a consent form and demographic queries to ensure eligibility and compliance with quotas. The background section introduced the EQ-5D-5L instrument and a five-question quiz designed to clarify attributes, address common misconceptions (e.g., scenarios involving “being in a coma”), and mitigate fraudulent participation. The choice task section included paired comparisons (EQ-5D-Y-3L vs. “being in a coma”; one warmup exercise and five tasks) and kaizen tasks (EQ-5D-Y-3L preference paths; one warmup and 20 tasks). The follow-up section featured one debriefing question, 31 items on socioeconomic status (SES), family, work, and health (including the PROMIS-16 instrument as it was described in the original article), and an open-text field for feedback on the survey experience. Overall, each respondent completed the EQ-5D-5L before the choice tasks and PROMIS-16 after the choice tasks as stand-alone instruments, with each item presented in a sequential one-item-per-screen format. Both instruments were administered using the official US English versions. Additional details on the full survey instrument are provided in Online Supplemental Appendix 1 of the protocol paper by Jumamyradov et al. [25].

It is also important to note that the data of this study was collected between 18 March and 6 April 2024 using the PROMIS-16 as it was described in the original article (published in February), not the erratum (published in August 2024; see Appendix 3) [12, 28]. The difference between the original article and the erratum includes the reversal of the two SLP items, such that the response options for SLP1 and SLP2 were swapped. In addition, the wording of SLP1, “I had problems during the day because of my sleep” (listed as SLP2 in the erratum), was revised to “I had problems during the day because of poor sleep” [28]. Additionally, the labels for two items (COG) are in the opposite order of the other PROMIS-16 items. For these two items, first and last response options represent the worst and best levels, respectively. In the item-testing survey, while the order of the eight dimensions remained the same, these items were shown alongside other similarly labeled COG items (not included in the PROMIS-16); however, our study included these items among other PROMIS-16 items, where first and last response options are the best and worst levels. Inconsistent label ordering may help reveal inattentive responding, as respondents must read and interpret each question rather than relying on straight-lining patterns [29].

### Statistical analyses

We first compare the PROMIS-16 measurement properties with those of the EQ-5D-5L using a series of psychometric analysis at the level of items and dimensions [24, 30]. Next, we performed an exploratory factor analysis to identify the combined structure of latent constructs. We used R version 4.4.2 to perform all statistical analyses in this study [31].

### Ceiling/floor percentages of the HRQoL constructs

The PROMIS-16 and EQ-5D-5L have eight and five dimensions, respectively. Dimensions that measured comparable HRQoL constructs (i.e. those sharing similar keywords or phrasing in their labels) were grouped into pairs, and McNemar’s test for paired proportions was used to assess differences in their ceiling and floor percentages.

Based on item descriptions, two conceptually matched pairs were identified. Specifically, EQ-5D-5L Mobility was aligned with PROMIS-16 PF, as PROMIS-16 PF items were derived from PROMIS Physical Function mobility subdomain. Additionally, EQ-5D-5L Anxiety/Depression was aligned with PROMIS-16 ANX and DEP. Within the PROMIS-16 items, we also grouped ANX with DEP and SLP with FTG. The ceiling percentage for two combined items is the percentage of respondents who select the best possible response on both items, while the floor percentage is the percentage who select the worst possible response on both items. Based on previous study of the PROMIS-29+2 and the EQ-5D-5L, we hypothesized that the PROMIS-16 items would show lower ceiling and higher floor percentages compared to its counterpart due to their 7-day recall period.[24]

### Informativity analysis of items

To evaluate informativity, Shannon’s Index (H’) and Shannon’s Evenness Index (J’) were computed for each item [32, 33]. For items with 5 response options, the Shannon Index (H’) ranges from 0 (i.e., all responses in one category) to a maximum of 2.32 (equal distribution across all response categories), with higher values indicating greater item informativity. The Shannon Evenness Index (J’) ranges from 0 to 1, with higher values reflecting more uniform distribution of responses across categories. The formulas used for calculating these indices are shown below:

$${\mathrm{Shannon}} {\mathrm{Index}} \left( {{\mathrm{H}}^{ \prime }} \right): {\mathrm{H}}^{ \prime } = - \mathop \sum \limits_{ i = 1}^{L} p_{i} \log_{2} p_{i} $$

$${\text{Shannon Evenness Index }}\left( {{\mathrm{J}}^{ \prime }} \right):{\mathrm{J}^{{\prime}}} = \frac{{{\mathrm{H}^{{\prime}}}}}{{\log_{2} {\mathrm{L}}}} $$

Here, L denotes the number of response categories and pᵢ represents the proportion of respondents choosing the *i*th response option [33]. We hypothesized that PROMIS-16 items would capture a wider spread of responses with less concentration at the extremes relative to the EQ-5D-5L due to difference in recall period [24].

### Validity assessment

To assess the relationship between items and self-reported health status (i.e., construct validity), we conducted linear regression analyses using EQ VAS as the outcome and incremental decrements in each item as predictors. For each item, we created four binary indicators representing whether a respondent reported a severity level greater than 1, 2, 3, or 4, respectively. We hypothesized significant negative associations between increasing severity levels along each item and EQ VAS. As a sensitivity analysis, we examined joint associations at the dimension level (e.g., simultaneously regressing PF1 and PF2 indicators on EQ VAS) and at the instrument level (e.g., including all PROMIS-16 indicators in a single regression model).

Polychoric correlation coefficients were calculated to assess the convergent and divergent validity among item pairs within and between the PROMIS-16 and EQ-5D-5L instruments. We examined three types of relationships: (1) cross-instrument correlations between PROMIS-16 and EQ-5D-5L, (2) within-instrument correlations among PROMIS-16 items, (3) within-instrument correlations among EQ-5D-5L items. Correlation strength was interpreted as very weak (< 0.20), weak (0.20–0.39), moderate (0.40–0.59), strong (0.60–0.79), and very strong (≥ 0.80) [34]. We hypothesized that item pairs of similar HRQoL constructs would demonstrate moderate to strong correlations.

### Structural validity

Using the polychoric correlation coefficients, EFA was employed to explore the latent structure underlying the PROMIS-16 and EQ-5D-5L instruments. The number of factors to retain was guided by parallel analysis [35, 36]. Given the assumption that HRQoL constructs within the two instruments are interrelated, an oblique rotation method (Oblimin) was used to enhance the clarity of factor interpretation [37]. Factor loadings with absolute values of 0.40 or higher were considered meaningful indicators of association between items and latent constructs [38, 39]. We hypothesized that conceptually aligned items from both instruments would load on similar factors, with the other PROMIS-16 items expected to load on new factors.

## Results

### Ceiling/floor percentages of the PROMIS-16 items

Table 1, Appendix 2a and 2b presents the PROMIS-16 items in the order that they appeared in the instrument. At the item level, the percentage of respondents endorsing the best level (ceiling) ranged from 27.9% (SLP2) to 72.9% (PF2), while the percentage endorsing the worst level (floor) ranged from 1.1% (PF1) to 24.7% (COG1). Among the floor percentages of these items, the two COG items showed notably high percentages.

**Table 1:** Ceiling and floor percentages of PROMIS-16 and EQ-5D-5L items, dimensions, and constructs

PROMIS-16						EQ-5D-5L			PROMIS-16 versus EQ-5D-5L	
Item	Ceiling, %	Floor, %	Dimension/construct	Ceiling, %	Floor, %	Item	Ceiling, %	Floor, %	Δ Ceiling, %	Δ Floor, %
PF1	68.0	1.1	Physical function	64.6	0.7	Mobility	79.9	0.4	− 15.3	0.3
PF2	72.9	2.3								
						Self-care	92.2	0.2		
						Usual activities	77.0	0.2		
						Pain/discomfort	42.7	0.8		
SOC1	61.7	1.2	Social roles and activities	50.8	0.8					
SOC2	54.9	3.0								
ANX1	45.0	2.4	Anxiety	34.4	2.1					
ANX2	37.6	4.1								
DEP1	37.2	4.0	Depression	35.5	2.5					
DEP2	48.0	3.3								
			Anxiety/depression	28.9	1.5	Anxiety/depression	44.7	3.5	− 15.8	− 2.0
SLP1	37.9	4.6	Sleep disturbance	23.2	3.1					
SLP2	27.9	7.8								
PI1	50.1	2.4	Pain interference	48.8	1.9					
PI2	64.0	3.1								
COG1	43.0	24.7	Cognitive function	28.9	19.7					
COG2	32.8	21.5								
FTG1	28.6	8.5	Fatigue	25.4	4.5					
FTG2	41.7	6.1								
			Sleep/fatigue	15.8	1.7					

The item content for PROMIS-16 and EQ-5D-5L is provided in Appendix 2a and Appendix 2b. The two items on sleep disturbance (SL1 and SL2) used the phrasing and item order from the original publication, not its erratum (Appendix 3). Anxiety / Depression and Sleep / Fatigue are HRQoL constructs composed of two of the eight dimensions of the PROMIS-16. All differences in Physical Function/Mobility and Anxiety / Depression were statistically significant according to McNemar’s test (*p* < 0.001)

### Ceiling/floor percentages of the EQ-5D-5L items

Table 1 shows the ceiling and floor percentages of the EQ-5D-5L items. Ceiling percentages ranged from 42.7% (Pain/Discomfort) to 92.2% (Self-care) while floor percentages ranged from 0.2% (Usual Activities and Self-care) to 3.5% (Anxiety/Depression).

Within the matched pairs, our hypothesis was partially supported: PROMIS-16 items showed significantly lower ceiling percentages, while PROMIS-16 Physical Function had higher floor percentages than EQ-5D-5L Mobility; however, PROMIS-16 Anxiety/Depression demonstrated lower floor percentages than EQ-5D-5L Anxiety/Depression (*p* < 0.001).

### Informativity analysis

Table 2 shows that the absolute informativity of the EQ-5D-5L items ranged from 0.48 for Self-care to 1.92 for Anxiety/Depression, while relative informativity ranged from 0.21 (Self-care) to 0.83 (Anxiety/Depression). PROMIS-16 items generally demonstrated higher informativity, with absolute values ranging from 1.30 (PF2) to 2.20 (COG2), and relative informativity ranging from 0.56 (PF2) to 0.95 (COG2). These findings supported our hypothesis that PROMIS-16 items exhibit greater informativity than their EQ-5D-5L counterparts.

**Table 2:** Informativity of PROMIS-16 and EQ-5D-5L items

PROMIS-16				EQ-5D-5L	
Item	Shannon’s index (H’)	Shannon’s Evenness index (J’)	Item	Shannon’s index (H’)	Shannon’s Evenness index (J’)
PF1	1.36	0.59	Mobility	0.98	0.42
PF2	1.30	0.56			
			Self-care	0.48	0.21
			Usual activities	1.04	0.45
			Pain/discomfort	1.69	0.73
SOC1	1.52	0.65			
SOC2	1.77	0.76			
ANX1	1.91	0.82	Anxiety/depression	1.92	0.83
ANX2	2.06	0.89			
DEP1	2.06	0.89			
DEP2	1.91	0.82			
SLP1	2.05	0.89			
SLP2	2.17	0.93			
PI1	1.78	0.77			
PI2	1.56	0.67			
COG1	2.03	0.87			
COG2	2.20	0.95			
FTG1	2.18	0.94			
FTG2	2.05	0.88			

The two items on sleep disturbance (SL1 and SL2) used the phrasing and item order from the original publication, not its erratum (Appendix 3)

PROMIS-16 dimensions: PF, physical function; SOC, social roles and activities; PI, pain interference; ANX, anxiety; DEP, depression; SLP, sleep disturbance; COG, cognitive function; FTG: fatigue

### Construct validity

Linear regression evidence confirmed our hypothesis: higher severity levels were associated with significantly lower EQ VAS scores (Table 3). Among PROMIS‑16 items, the steepest first‑step declines were observed for PF1 (− 10.11, *p* < 0.001) and PF2 (− 10.58, *p* < 0.001) while among EQ‑5D‑5L items, Mobility (− 13.44, *p* < 0.001), Self-care (− 20.76, *p* < 0.001) and Usual Activities (− 13.19, *p* < 0.001) showed the largest drops. In contrast, smaller or non-significant changes were observed at the highest severity level for items such as PF1, PF2, SOC1, COG1, COG2, Mobility, Self-care, and Usual Activities. Notably, PROMIS-16 cognitive items (COG1, COG2) and EQ-5D-5L Mobility, Self-care showed large positive transitions from Level 4 to 5. Appendix 4 shows that in multivariate models (dimension‑, construct‑, and instrument‑level), effect sizes diminished, and many steps changed sign or lost significance, indicating substantial overlap among items; at the instrument level, EQ‑5D‑5L retained stronger independent associations than PROMIS‑16.

**Table 3:** Item-level regression results between EQ VAS and PROMIS-16 and EQ-5D-5L items

			Level 1 to 2		Level 2 to 3		Level 3 to 4		Level 4 to 5	
Items	Intercept	*p* value	β	*p* value	β	*p* value	β	*p* value	β	p-value
PROMIS-16										
PF1	79.54	< 0.001	− 10.11	< 0.001	− 9.56	< 0.001	− 11.08	< 0.001	4.70	0.183
PF2	79.18	< 0.001	− 10.58	< 0.001	− 8.66	< 0.001	− 7.65	< 0.001	− 3.00	0.262
SOC1	79.62	< 0.001	− 7.35	< 0.001	− 10.41	< 0.001	− 3.59	0.091	− 3.26	0.365
SOC2	80.96	< 0.001	− 7.26	< 0.001	− 7.49	< 0.001	− 8.15	< 0.001	− 7.64	< 0.001
ANX1	80.72	< 0.001	− 5.64	< 0.001	− 6.33	< 0.001	− 5.14	< 0.001	− 10.74	< 0.001
ANX2	80.90	< 0.001	− 4.40	< 0.001	− 5.28	< 0.001	− 5.94	< 0.001	− 8.15	< 0.001
DEP1	81.40	< 0.001	− 4.94	< 0.001	− 5.49	< 0.001	− 6.44	< 0.001	− 10.50	< 0.001
DEP2	80.19	< 0.001	− 4.92	< 0.001	− 5.88	< 0.001	− 7.55	< 0.001	− 6.33	0.003
SLP1	81.14	< 0.001	− 5.22	< 0.001	− 4.87	< 0.001	− 5.91	< 0.001	− 7.82	< 0.001
SLP2	82.05	< 0.001	− 4.88	< 0.001	− 5.01	< 0.001	− 5.51	< 0.001	− 8.22	< 0.001
PI1	81.34	< 0.001	− 7.67	< 0.001	− 8.59	< 0.001	− 7.61	< 0.001	− 11.36	< 0.001
PI2	79.87	< 0.001	− 8.41	< 0.001	− 7.41	< 0.001	− 7.76	< 0.001	− 6.41	0.005
COG1	75.97	< 0.001	− 5.37	< 0.001	− 5.68	< 0.001	1.28	0.459	**14.89**	< 0.001
COG2	77.83	< 0.001	− 5.89	< 0.001	− 5.27	< 0.001	0.16	0.911	**14.61**	< 0.001
FTG1	84.03	< 0.001	− 7.69	< 0.001	− 5.06	< 0.001	− 5.10	< 0.001	− 8.49	< 0.001
FTG2	81.49	< 0.001	− 6.30	< 0.001	− 6.36	< 0.001	− 3.99	0.002	− 5.26	0.002
EQ-5D-5L										
Mobility	78.34	< 0.001	− 13.44	< 0.001	− 11.25	< 0.001	− 13.86	< 0.001	**23.57**	< 0.001
Self-care	76.51	< 0.001	− 20.76	< 0.001	− 7.80	0.007	− 20.75	0.009	**22.80**	0.044
Usual activities	78.97	< 0.001	− 13.19	< 0.001	− 13.81	< 0.001	− 17.42	< 0.001	6.84	0.370
Pain/discomfort	82.58	< 0.001	− 8.49	< 0.001	− 11.50	< 0.001	− 15.85	< 0.001	− 11.48	0.003
Anxiety/depression	81.46	< 0.001	− 6.68	< 0.001	− 5.44	< 0.001	− 11.19	< 0.001	− 8.27	< 0.001

The two items on sleep disturbance (SL1 and SL2) used the phrasing and item order from the original publication, not its erratum (Appendix 3). We regressed the item responses as indicator variables on EQ VAS by item. Each row was a separate estimation. For example, the PF1 regression (row 1) had an intercept and four indicator variables representing four incremental decrements of PF1. The bolded coefficients are positive and significant (*p*-value < 0.05)

PROMIS-16 dimensions: PF, physical function; SOC, social roles and activities; PI, pain interference; ANX, anxiety; DEP, depression; SLP, sleep disturbance; COG, cognitive function; FTG: fatigue

### Convergent and divergent validity

Table 4 illustrates the polychoric’s correlation coefficients between PROMIS-16 and EQ-5D-5L items. Items measuring the same construct showed strong correlations, namely PF1, PF2 with Mobility and ANX1, ANX2, DEP1, DEP2 with Anxiety/Depression. Additionally, among the other items, PF1 and PF2 also demonstrated moderate to strong correlations with Self-care, Usual Activities, and Pain/Discomfort. COG showed very weak or no correlations with any EQ-5D-5L items, whereas among the five EQ-5D-5L dimensions, SLP and FTG were most strongly correlated with Anxiety/Depression. Appendix 5 and Appendix 6 present the polychoric correlations among PROMIS-16 items and EQ-5D-5L items, respectively. As expected, the ANX/DEP and SLP/FTG constructs within the PROMIS-16 demonstrated strong inter-item correlations. In contrast, the COG items showed very weak to no correlations with other PROMIS-16 items. The Anxiety/Depression item of the EQ-5D-5L exhibited weak to moderate correlations with other EQ-5D-5L items.

**Table 4:**
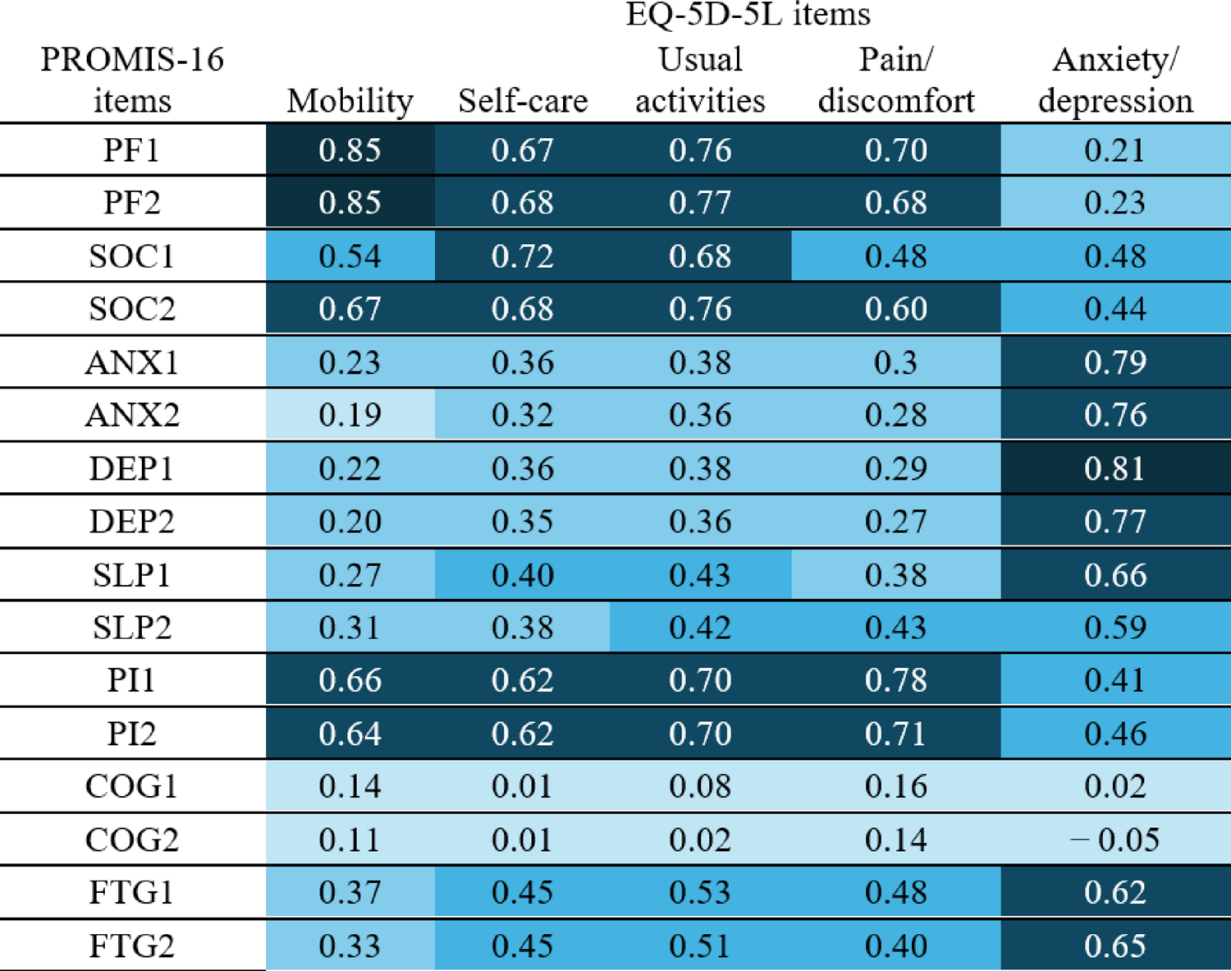
Polychoric Correlation Coefficients between PROMIS-16 and EQ-5D-5L items

The two items on sleep disturbance (SL1 and SL2) used the phrasing and item order from the original publication, not its erratum (Appendix 3). Cell colors indicate correlation strength: very weak (< 0.20, very light blue), weak (0.20–0.39, light blue), moderate (0.40–0.59, blue), strong (0.60–0.79, dark blue), and very strong (≥ 0.80, very dark blue). Within-instrument correlations are shown in Appendices 6 and 7

PROMIS-16 dimensions: PF, physical function; SOC, social roles and activities; PI, pain interference; ANX, anxiety; DEP, depression; SLP, sleep disturbance; COG, cognitive function; FTG: fatigue

### Structural validity

Exploratory factor analysis identified six distinct factors from the PROMIS-16 and EQ-5D-5L polychoric correlations (Table 5, Appendix 5 and 7). Aligning with our hypothesis, PROMIS-16 and EQ-5D-5L items measuring the same HRQoL constructs loaded onto the same factors, which are physical functioning (PF1, PF2, Mobility) and Anxiety / Depression (Anxiety/Depression, ANX1, ANX2, DEP1, DEP2). Notably, the physical functioning factor was broader than suggested by the pairing of Mobility with PF, as it also included Self-care, Usual Activities, and Pain/Discomfort. The remaining PROMIS-16 items formed four additional factors: SOC, PI, COG, and SLP / FTG. Most items demonstrated good fit with low uniqueness values (≤ 0.60), except for SOC2. The COG factor was not correlated with any other factors (Table 6), and the PI factor showed negative correlations with all other factors.

**Table 5:** PROMIS-16 and EQ-5D-5L factor loadings and uniqueness of items

	Factor loadings						
Items	1. Physical function	2. social role & activities	3. Anxiety/depression	4. Pain Interference	5. Cognitive Function	6. Sleep/fatigue	Uniqueness
PROMIS-16							
PF1	0.87						0.23
PF2	0.85						0.20
SOC1		0.90					0.19
SOC2		0.42					0.64
ANX1			0.88				0.22
ANX2			0.89				0.20
DEP1			0.94				0.12
DEP2			0.90				0.19
SLP1						0.70	0.46
SLP2						0.67	0.52
PI1				1.01			− 0.02
PI2				0.71			0.46
COG1					0.97		0.06
COG2					0.91		0.17
FTG1						0.93	0.12
FTG2						0.83	0.30
EQ-5D-5L							
Mobility	0.98						0.03
Self-care	0.61						0.55
Usual activities	0.69						0.48
Pain/discomfort	0.53						0.55
Anxiety/depression			0.85				0.30

The two items on sleep disturbance (SL1 and SL2) used the phrasing and item order from the original publication, not its erratum (Appendix 3). Factor loadings < 0.40 were suppressed

PROMIS-16 dimensions: PF, physical function; SOC, social roles and activities; PI, pain interference; ANX, anxiety; DEP, depression; SLP, sleep disturbance; COG, cognitive function; FTG: fatigue

**Table 6:**
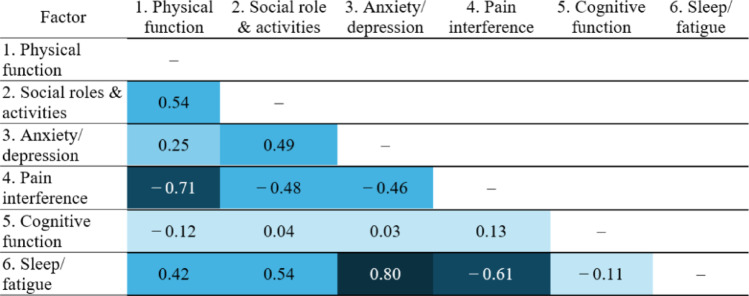
PROMIS-16 and EQ-5D-5L factor correlation matrix

Cell colors indicate correlation strength, |r|: very weak (< 0.20, very light blue), weak (0.20–0.39, light blue), moderate (0.40–0.59, blue), strong (0.60–0.79, dark blue), and very strong (≥ 0.80, very dark blue)

## Discussion

### Comparing the PROMIS-16 stand-alone instrument and item-testing evidence

Currently, six studies have thus far examined the PROMIS-16 items; however, each embedded these items in a broader item set (PROMIS-29+2, PROPr-14, UPMC16, SIGNAL). To the best of our knowledge, this survey is the first to include the PROMIS-16 as a stand-alone instrument, so we begin this discussion by comparing the response distributions from the stand-alone instrument with those reported in the original item-testing survey (Appendices 2a, 2b, 3 and 7) [12].

Compared to the original item-testing survey, the stand-alone version showed substantially higher ceiling percentages for the SOC items, with increases of more than 20 percentage points for SOC1 and over 10 points for SOC2 (*p* < 0.001). This pattern is consistent with prior PROMIS research indicating that broader item sets and adaptive administrations are associated with lower floor and ceiling percentages, whereas fixed short forms with more limited severity coverage may exhibit higher ceiling percentages [40]. Furthermore, the PF and SOC items are the only items without a seven-day recall period, and their proximity in the stand-alone instrument may have contributed to the greater similarity in response distributions compared to the item-testing survey.

We also observed that the floor percentages for the COG items were markedly higher in the stand-alone instrument, with increases of 18 and 17 percentage points for COG1 and COG2, respectively (*p* < 0.001). These items are reverse-framed relative to most PROMIS dimensions. In the original item-testing survey, consistent presentation and the appearance alongside three other COG items likely helped mitigate confusion. In the stand-alone administration, the COG items were administered later in a survey that included extensive valuation tasks, which may have increased respondent fatigue or satisficing. Under such conditions, items with reversed response order may be more likely to reveal inattentive responding, potentially contributing to the differences in floor percentages [29].

Overall, the PROMIS-16 as a stand-alone instrument produces higher proportions of best SOC and worst COG responses compared to item-testing survey. Given that ceiling and floor percentages also depend on external factors (e.g., sample characteristics), these differences should be interpreted as potential variability rather than to attribute differences to a single cause. At the same time, this pattern is an important point and should be confirmed prior to future comparative studies and potentially addressed in a modified version of the PROMIS-16 instrument.

### Comparing the PROMIS-16 and EQ-5D-5L

PROMIS-16 and EQ-5D-5L differ in terms of construct coverage, response scales, recall period and measurement performance. While both instruments share common constructs such as Physical Function and Anxiety/Depression, PROMIS-16 captures a broader range of HRQoL aspects and provides more detailed information within overlapping constructs, as reflected in its lower ceiling percentages and higher informativity scores compared to EQ-5D-5L items within the same matched pairs. One possible reason for these differences may be the recall period embedded in some PROMIS-16 items but not in EQ-5D-5L, a factor previously highlighted by Rencz et al. [24] Except for PF and SOC items, PROMIS-16 items ask respondents to reflect on their experiences over the past 7 days, whereas EQ-5D-5L focuses on the respondent’s health "today." The longer recall window in PROMIS-16 may reduce the likelihood of respondents over- or understating their current condition based on temporary fluctuations, potentially explaining the reduced ceiling percentages and greater informativity [24, 41]. However, we acknowledge that previous research has shown that the recall period may have a limited impact on PROM responses, particularly for PROMIS physical functioning measures [42, 43]. Another potential explanation is that the PROMIS-16 has two items per dimension and this increased respondent burden naturally leads to gains in precision. To achieve similar gains, splitting EQ-5D-5L dimensions in specific circumstances and the addition of bolt-ons could be considered as future research directions [44–46]. In addition, differences in response format and item wording may also contribute to the higher informativity observed for PROMIS-16, as prior research suggests that these features can influence measurement range captured by an instrument [47].

Regarding construct validity, most PROMIS-16 and EQ-5D-5L items performed as expected at the item level, with increasing severity levels corresponding to lower EQ VAS scores. In some cases, exceptions (i.e., large positive transition steps in COG, Mobility and Self-care) may reflect respondents’ adaptation to chronic or age-related conditions, wherein individuals adjust expectations or develop coping strategies over time. However, for the Cognitive Function items, the sharp increase in EQ VAS scores between levels 4 and 5, combined with high floor percentages, once again suggests that inattentive responding may have influenced responses. At the instrument level, EQ-5D-5L aligned more closely with respondents’ overall health ratings than PROMIS-16. This is likely because EQ-5D-5L’s concise structure captures core health dimensions that respondents intuitively consider when assessing their general health and the EQ VAS was administered right after the EQ-5D-5L. In contrast, PROMIS-16 encompasses broader and less immediately visible constructs, such as Cognitive Function, Sleep/Fatigue, and Social roles & Activities, that may be perceived as less central to overall health, thereby weakening its association with EQ VAS. On the other hand, given that EQ VAS is part of the EQ-5D-5L and was administered following this instrument, future research would benefit from comparing these instruments using external comparators administered independently.

From the convergent validity and structural assessments, several noteworthy findings emerged. First, PROMIS-16 contributed additional health dimensions beyond those captured by the EQ-5D-5L. While COG was uniquely represented and thus aligning well with the widespread use of the EQ-5D-5L Cognition bolt-ons [48, 49], SOC and SLP/FTG, though correlated with EQ-5D-5L dimensions, loaded onto distinct factors, which suggests that they capture related but separable aspects of health.

Second, we observed a negative correlation between the Pain Interference factor and other factors. These do not mean negative relationships between items; instead, it indicates that individuals who score higher on the Pain Interference factor tend to score lower on others, after accounting for the shared variance structure imposed by rotation. One possible explanation is the different way these items were constructed: PI1 and PI2 ask how much a person’s experience of pain disrupts or interferes with their daily activities, rather than measuring pain or discomfort intensity itself. In other words, some level of functioning is needed to indicate interference with functioning, and the degree of interference depends on an individual’s perception of causality.

Third, the EFA showed that SOC2 had a much lower factor loading (0.42) and higher uniqueness (> 0.60) compared to SOC1 (0.90) within the Social Roles & Activities factor. This may reflect differences in item content: SOC1 addresses broad, routine responsibilities (“I have trouble taking care of my regular personal responsibilities…”), while SOC2 is more complex and context-dependent (“I have trouble doing all of the activities with friends that I want to do…”), potentially leading to more varied interpretations across respondents.

Fourth, although Edelen et al. acknowledged that deriving the PROMIS-16 PF items solely from the PROMIS Physical Function mobility subdomain was a limitation [12], our findings indicate that these items share substantial variance with broader aspects of physical functioning, including Self-Care, Usual Activities, and Pain/Discomfort. This suggests that, in general population samples, PROMIS-16 PF items may reflect broader aspects of physical functioning rather than mobility alone. Nevertheless, the restricted concept range of these two items should be considered when interpreting results. Future research should examine the performance of them in disease-specific populations where physical functioning is a central outcome, such as cardiovascular or rheumatologic conditions.

Together, these findings highlight opportunities for future research to refine both instruments. For EQ-5D-5L, further development of bolt-ons capturing dimensions such as cognition, sleep, and fatigue could enhance its conceptual coverage in specific populations where these are particularly relevant, while for PROMIS-16, further development and refinement of items, particularly in how questions of the items should be constructed in the same patterns (e.g. recall period, framing direction, item complexity), may improve measurement precision and structural validity.

While our study offers meaningful insights into the PROMIS-16 and EQ-5D-5L instruments, there remains room for improvement in future research. First, similar to the studies by Edelen et al. and Hays et al., our analysis relied on a general population sample drawn from an online survey panel as PROMIS-16 is a generic HRQoL instrument and should be able to demonstrate adequate measurement properties across the full health spectrum [12, 16]. This approach may introduce selection bias due to variation in digital literacy and internet access, and may partially explain the excessively high ceiling percentages observed in some items and dimensions. Additionally, since these findings are based on a general population sample, they may not generalize to clinical populations. Instruments that appear to provide less information or exhibit higher ceiling percentages in relatively healthy samples may nonetheless demonstrate greater sensitivity in clinical populations with higher levels of impairment. As such, the observed differences in measurement properties between the PROMIS-16 and EQ-5D-5L should be interpreted as sample-dependent and may differ when these instruments are applied in specific patient populations. Future studies should consider recruiting disease-specific populations and employing more inclusive administration methods (e.g., research nurse-assisted surveys) to more accurately evaluate the reliability and validity of the PROMIS-16 across diverse populations. Second, we recognize that the sample was recruited through a commercial online panel (Dynata), which might pose data quality limitations [50]. Although quality control measures were implemented to mitigate these concerns, future research would benefit from using more representative sampling platforms.

Differences in sample size between our survey and the PROMIS item-testing studies may have contributed to observed distributional differences. The larger sample in Edelen et al. likely produced more stable estimates, while our smaller sample may have amplified fluctuations that would not replicate in a larger cohort [12]. In addition, the five-question quiz at the start of the survey may have influenced how respondents approached the EQ-5D-5L items. As such, future research should randomize instrument order to better isolate framing, cognitive burden, and response bias. Last but not least, because our data were collected between March and April 2024, prior to the publication of the August 2024 erratum, we used the original version of the PROMIS-16 SLP items, which may have influenced their performance. Moreover, possible unknown differences in the representativeness of the original PROMIS-16 survey sample compared to our survey may also have contributed to observed differences.

## Conclusion

This study found that PROMIS-16 items demonstrated broader conceptual coverage and greater informativity than EQ-5D-5L, capturing distinct dimensions such as Cognitive Function, and Sleep/Fatigue. However, we also identified issues with inconsistent item framing and potential response biases, particularly in the Cognitive Function items, that may impact the validity of the PROMIS-16. Refining PROMIS-16 item design (e.g. consistent labeling order, recall period, item complexity) and developing bolt-ons for EQ-5D-5L for specific populations could improve measurement precision and conceptual coverage, enhancing their application in research and clinical settings. This comparative evidence may help researchers faced with the choice between these alternative HRQOL instruments for their future studies and those looking to enhance these instruments [44, 51, 52].

## Supplementary Information

Below is the link to the electronic supplementary material.

Supplementary Material 1

## Data Availability

Data will be made available upon reasonable request.

## References

[1] Marshall, S., Haywood, K., & Fitzpatrick, R. (2006). Impact of patient-reported outcome measures on routine practice: A structured review. *Journal of Evaluation in Clinical Practice,**12*, 559–568. 10.1111/j.1365-2753.2006.00650.x 16987118 10.1111/j.1365-2753.2006.00650.x

[2] Black, N. (2013). Patient reported outcome measures could help transform healthcare. *British Medical Journal Open*. 10.1136/bmj.f167 10.1136/bmj.f167 23358487

[3] Dawson, J., Doll, H., Fitzpatrick, R., Jenkinson, C., & Carr, A. J. (2010). The routine use of patient reported outcome measures in healthcare settings. *British Medical Journal Open,**340*, c186. 10.1136/bmj.c186 10.1136/bmj.c186 20083546

[4] Bull, C., Teede, H., Watson, D., & Callander, E. J. (2022). Selecting and implementing patient-reported outcome and experience measures to assess health system performance. *JAMA Health Forum,**3*, Article e220326. 10.1001/jamahealthforum.2022.0326 36218960 10.1001/jamahealthforum.2022.0326

[5] Kingsley, C., & Patel, S. (2017). Patient-reported outcome measures and patient-reported experience measures. *BJA Education,**17*, 137–144. 10.1093/bjaed/mkw060

[6] Cella, D., Riley, W., Stone, A., Rothrock, N., Reeve, B., Yount, S., et al. (2010). The Patient-Reported Outcomes Measurement Information System (PROMIS) developed and tested its first wave of adult self-reported health outcome item banks: 2005–2008. *Journal of Clinical Epidemiology,**63*, 1179–1194. 10.1016/j.jclinepi.2010.04.011. Elsevier; 20685078 10.1016/j.jclinepi.2010.04.011 PMC2965562

[7] Evans, J. P., Smith, A., Gibbons, C., Alonso, J., & Valderas, J. M. (2018). The National Institutes of Health Patient-Reported Outcomes Measurement Information System (PROMIS): A view from the UK. *Patient Related Outcome Measures,**9*, 345–352. 10.2147/PROM.S141378. Dove Press; 30498382 10.2147/PROM.S141378 PMC6207259

[8] Rencz, F., Gulácsi, L., Drummond, M., Golicki, D., Prevolnik Rupel, V., Simon, J., et al. (2016). EQ-5D in Central and Eastern Europe: 2000–2015. *Quality of Life Research,**25*, 2693–2710. 10.1007/s11136-016-1375-6 27472992 10.1007/s11136-016-1375-6

[9] Kennedy-Martin, M., Slaap, B., Herdman, M., van Reenen, M., Kennedy-Martin, T., Greiner, W., et al. (2020). Which multi-attribute utility instruments are recommended for use in cost-utility analysis? A review of national health technology assessment (HTA) guidelines. *European Journal of Health Economics,**21*, 1245–1257. 10.1007/s10198-020-01195-8 10.1007/s10198-020-01195-8 PMC7561556 32514643

[10] Wang, A., Rand, K., Yang, Z., Brooks, R., & Busschbach, J. (2022). The remarkably frequent use of EQ-5D in non-economic research. *European Journal of Health Economics,**23*, 1007–1014. 10.1007/s10198-021-01411-z 10.1007/s10198-021-01411-z PMC9304056 34846623

[11] Devlin, N. J., Xie, F., Slaap, B., & Stolk, E. (2025). Measuring and Valuing Health Using EuroQol Instruments: New Developments 2025 and Beyond. *Applied Health Economics and Health Policy*. 10.1007/s40258-025-00989-2 40705271 10.1007/s40258-025-00989-2 PMC12535937

[12] Edelen, M. O., Zeng, C., Hays, R. D., Rodriguez, A., Hanmer, J., Baumhauer, J., et al. (2024). Development of an ultra-short measure of eight domains of health-related quality of life for research and clinical care: the patient-reported outcomes measurement information system® PROMIS®-16 profile. *Quality of Life Research*. 10.1007/s11136-023-03597-6 38319489 10.1007/s11136-023-03597-6 PMC11800902

[13] Edelen, M. O., Hays, R. D., & Herman, P. M. (2025). Introducing the PROMIS-16 profile 1.0. *Quality of Life Research,**34*, 1–2. 10.1007/s11136-024-03885-9 39831935 10.1007/s11136-024-03885-9 PMC11802287

[14] Cella, D., Choi, S. W., Condon, D. M., Schalet, B., Hays, R. D., Rothrock, N. E., et al. (2019). PROMIS® Adult Health Profiles: Efficient short-form measures of seven health domains. *Value in Health,**22*, 537–544. 10.1016/j.jval.2019.02.004. Elsevier; 31104731 10.1016/j.jval.2019.02.004 PMC7201383

[15] Hays, R. D., Bjorner, J. B., Revicki, D. A., Spritzer, K. L., & Cella, D. (2009). Development of physical and mental health summary scores from the patient-reported outcomes measurement information system (PROMIS) global items. *Quality of Life Research,**18*, 873–880. 10.1007/s11136-009-9496-9 19543809 10.1007/s11136-009-9496-9 PMC2724630

[16] Hays, R. D., Herman, P. M., Rodriguez, A., Slaughter, M., Zeng, C., & Edelen, M. O. (2025). The PROMIS-16 reproduces the PROMIS-29 physical and mental health summary scores accurately in a probability-based internet panel. *Quality of Life Research*. 10.1007/s11136-024-03662-8 38652369 10.1007/s11136-024-03662-8 PMC11496378

[17] Hanmer, J., Zeng, C., Cizik, A. M., Raad, J. H., Tsevat, J., Rodriguez, A., et al. (2025). Agreement of PROMIS Preference (PROPr) scores generated from the PROMIS-29 + 2 and the PROMIS-16. *Quality of Life Research,**34*, 43–51. 10.1007/s11136-024-03827-5 39508976 10.1007/s11136-024-03827-5 PMC11802291

[18] Zeng, C., Hays, R. D., Rodriguez, A., Hanmer, J., Herman, P. M., & Edelen, M. O. (2025). Comparing patient-reported outcomes measurement information system® (PROMIS®)-16 domain scores with the PROMIS-29 and 5-item PROMIS cognitive function scores. *Quality of Life Research,**34*, 27–34. 10.1007/s11136-024-03747-4 39143447 10.1007/s11136-024-03747-4 PMC11802290

[19] Rodriguez, A., Zeng, C., Hays, R. D., Herman, P. M., & Edelen, M. O. (2025). Longitudinal validation of the PROMIS-16 in a sample of adults in the United States with back pain. *Quality of Life Research,**34*, 35–42. 10.1007/s11136-024-03826-6 39505759 10.1007/s11136-024-03826-6 PMC11802292

[20] EuroQol - a new facility for the measurement of health-related quality of life. Health Policy. 1990;16:199–208. 10.1016/0168-8510(90)90421-9 10.1016/0168-8510(90)90421-9 10109801

[21] Devlin, N. J., & Brooks, R. (2017). EQ-5D and the EuroQol Group: Past, Present and Future. *Applied Health Economics and Health Policy,**15*, 127–137. 10.1007/s40258-017-0310-5 28194657 10.1007/s40258-017-0310-5 PMC5343080

[22] Brooks, R. (1996). EuroQol: The current state of play. *Health Policy,**37*, 53–72. 10.1016/0168-8510(96)00822-6 10158943 10.1016/0168-8510(96)00822-6

[23] Herdman, M., Gudex, C., Lloyd, A., Janssen, M. F., Kind, P., Parkin, D., et al. (2011). Development and preliminary testing of the new five-level version of EQ-5D (EQ-5D-5L). *Quality of Life Research,**20*, 1727–1736. 10.1007/s11136-011-9903-x 21479777 10.1007/s11136-011-9903-x PMC3220807

[24] Rencz, F., Brodszky, V., & Janssen, M. F. (2023). A Direct Comparison of the Measurement Properties of EQ-5D-5L, PROMIS-29+2 and PROMIS Global Health Instruments and EQ-5D-5L and PROPr Utilities in a General Population Sample. *Value Health.,**26*, 1045–1056. 10.1016/j.jval.2023.02.002 36804583 10.1016/j.jval.2023.02.002

[25] Jumamyradov, M., Craig, B. M., Rivero-Arias, O., & Jakubczyk, M. (2023). Child health valuation protocol for a discrete choice experiment comparing paired comparison and kaizen tasks and estimating US EQ-5D-Y-3L values on an experience scale. *British Medical Journal Open,**13*, e077256. 10.1136/bmjopen-2023-077256 10.1136/bmjopen-2023-077256 PMC10603523 37879694

[26] Jumamyradov, M., Craig, B. M., & Jakubczyk, M. (2025). Revisiting the Valuation of Child Health-Related Quality of Life: Replacing Paired Comparisons With Kaizen Tasks and QALY Scaling With Experience Scaling. *Medical Care.,**63*, 771. 10.1097/MLR.0000000000002200 40846650 10.1097/MLR.0000000000002200 PMC12422609

[27] Jumamyradov, M., & Craig, B. M. (2025). Measuring Effectiveness Based on Patient Experience (Instead of QALYs) in US Value Assessments. *PharmacoEconomics,**43*, 171–176. 10.1007/s40273-024-01444-1 39487899 10.1007/s40273-024-01444-1 PMC11782394

[28] Edelen, M. O., Zeng, C., Hays, R. D., Rodriguez, A., Hanmer, J., Baumhauer, J., et al. (2025). Correction: Development of an ultra-short measure of eight domains of health-related quality of life for research and clinical care: The patient-reported outcomes measurement information systemⓇ PROMISⓇ-16 profile. *Quality of Life Research,**34*, 17–18. 39192142 10.1007/s11136-024-03762-5 PMC11802702

[29] van Sonderen, E., Sanderman, R., & Coyne, J. C. (2013). Ineffectiveness of Reverse Wording of Questionnaire Items: Let’s Learn from Cows in the Rain. *PLoS ONE,**8*, e68967. 10.1371/journal.pone.0068967 23935915 10.1371/journal.pone.0068967 PMC3729568

[30] Brazier, J., & Deverill, M. (1999). A checklist for judging preference-based measures of health related quality of life: Learning from psychometrics. *Health Economics,**8*, 41–51. 10082142 10.1002/(sici)1099-1050(199902)8:1<41::aid-hec395>3.0.co;2-#

[31] R: The R Project for Statistical Computing [Internet]. [cited 2025 Jun 5]. https://www.r-project.org/. Accessed 5 Jun 2025

[32] Shannon, C. E. (1948). A mathematical theory of communication. *Bell System Technical Journal,**27*, 379–423. 10.1002/j.1538-7305.1948.tb01338.x

[33] Janssen, M. F., Birnie, E., & Bonsel, G. J. (2007). Evaluating the discriminatory power of EQ-5D, HUI2 and HUI3 in a US general population survey using Shannon’s indices. *Quality of Life Research,**16*, 895–904. 10.1007/s11136-006-9160-6 17294285 10.1007/s11136-006-9160-6 PMC1915610

[34] Evans, J.D. (1996). Straightforward statistics for the behavioral sciences. Belmont, CA, US: Thomson Brooks/Cole Publishing Co; 1996. p. xxii, 600.

[35] Howard, M. C. (2016). A review of exploratory factor analysis decisions and overview of current practices: What we are doing and how can we improve? *Int J Human–Computer Interact,**32*, 51–62. 10.1080/10447318.2015.1087664

[36] Ciobanu, L. G., Stankov, L., Ahmed, M., Heathcote, A., Clark, S. R., & Aidman, E. (2023). Multifactorial structure of cognitive assessment tests in the UK Biobank: A combined exploratory factor and structural equation modeling analyses. *Frontiers in Psychology,**14*, Article 1054707. 10.3389/fpsyg.2023.1054707 36818106 10.3389/fpsyg.2023.1054707 PMC9937787

[37] Costello, A. B., Osborne, J. (2025). Best practices in exploratory factor analysis: four recommendations for getting the most from your analysis. University of Massachusetts Amherst; [cited 2025 May 29]; 10.7275/JYJ1-4868

[38] Hinkin, T. R. (1995). A review of scale development practices in the study of organizations. *Journal of Management,**21*, 967–988. 10.1016/0149-2063(95)90050-0

[39] Hinkin, T. R. (1998). A brief tutorial on the development of measures for use in survey questionnaires. *Organizational Research Methods,**1*, 104–121. 10.1177/109442819800100106

[40] Alhasani, R., Ataman, R., Nafees, Z., Luong, A., Auneau-Enjalber, L., Quigley, A., et al. (2025). Enhancing Interpretability of Patient Reported Outcome Measurement Information System (PROMIS) and Related Measures in Rehabilitation Populations: A Systematic Review of Clinical and Research Applications. *Archives of Physical Medicine and Rehabilitation*. 10.1016/j.apmr.2025.11.017 41338482 10.1016/j.apmr.2025.11.017

[41] Peasgood, T., Caruana, J. M., & Mukuria, C. (2023). Systematic review of the effect of a one-day versus seven-day recall duration on patient reported outcome measures (PROMs). *The Patient - Patient-Centered Outcomes Research,**16*, 201–221. 10.1007/s40271-022-00611-w 36786931 10.1007/s40271-022-00611-w PMC10121527

[42] Peipert, J. D., Chapman, R., Shaunfield, S., Kallen, M. A., Schalet, B. D., & Cella, D. (2022). Do You Recall?: Results From a Within-Person Recall Study of the Patient-Reported Outcomes Measurement Information System (PROMIS) Short Form v2.0—Physical Function 8c. *Value in Health,**25*, 161–166. 10.1016/j.jval.2021.08.011 35094787 10.1016/j.jval.2021.08.011

[43] Condon, D. M., Chapman, R., Shaunfield, S., Kallen, M. A., Beaumont, J. L., Eek, D., et al. (2020). Does recall period matter? Comparing PROMIS® physical function with no recall, 24-hr recall, and 7-day recall. *Quality of Life Research,**29*, 745–753. 10.1007/s11136-019-02344-0 31701432 10.1007/s11136-019-02344-0 PMC7199782

[44] Rencz, F., & Janssen, M. F. (2024). Testing the psychometric properties of 9 bolt-ons for the EQ-5D-5L in a general population sample. *Value in Health,**27*, 943–954. 10.1016/j.jval.2024.03.2195 38599517 10.1016/j.jval.2024.03.2195

[45] Rencz, F., & Janssen, M. F. (2022). Analyzing the pain/discomfort and anxiety/depression composite domains and the meaning of discomfort in the EQ-5D: A mixed-methods study. *Value in Health,**25*, 2003–2016. 10.1016/j.jval.2022.06.012 35973925 10.1016/j.jval.2022.06.012

[46] Pham, M., Craig, B. M., & Rencz, F. (2025). The psychometric performance of the EQ-5D-5L composite and component items in the U.S. general population and by age group. *Quality of Life Research*. 10.1007/s11136-025-04053-3 40911276 10.1007/s11136-025-04053-3 PMC12689771

[47] Liegl, G., Gandek, B., Fischer, H. F., Bjorner, J. B., Ware, J. E., Rose, M., et al. (2017). Varying the item format improved the range of measurement in patient-reported outcome measures assessing physical function. *Arthritis Research & Therapy,**19*, Article 66. 10.1186/s13075-017-1273-5 28320462 10.1186/s13075-017-1273-5 PMC5359818

[48] Rencz, F., Pangestu, S., Mulhern, B., Finch, A. P., & Janssen, M. F. (2025). Development and use of cognition bolt-ons for the EQ-5D-3L and EQ-5D-5L: A systematic review. *Value in Health*. 10.1016/j.jval.2025.05.015 40490133 10.1016/j.jval.2025.05.015

[49] Rencz, F., Pangestu, S., Mulhern, B., Finch, A. P., & Janssen, M. F. (2025). Psychometric Properties of Cognition Bolt-Ons for the EQ-5D-3L and EQ-5D-5L: A Systematic Review. *Value Health J Int Soc Pharmacoeconomics Outcomes Res.,**S1098–3015*(25), 05610–05614. 10.1016/j.jval.2025.09.3053 10.1016/j.jval.2025.09.3053 41046102

[50] Peer, E., Rothschild, D., Gordon, A., Evernden, Z., & Damer, E. (2022). Data quality of platforms and panels for online behavioral research. *Behavior Research Methods,**54*, 1643–1662. 10.3758/s13428-021-01694-3 34590289 10.3758/s13428-021-01694-3 PMC8480459

[51] Finch, A. P., Brazier, J., & Mukuria, C. (2021). Selecting Bolt-on Dimensions for the EQ-5D: Testing the Impact of Hearing, Sleep, Cognition, Energy, and Relationships on Preferences Using Pairwise Choices. *Medical Decision Making,**41*, 89–99. 10.1177/0272989X20969686 33256502 10.1177/0272989X20969686 PMC7780267

[52] Xu, R. H., Rencz, F., Sun, R., Dong, D., & Zhang, S. (2025). Development and testing of the psychometric properties of 20 bolt-on items for the EQ-5D-5L across 31 rare diseases. *Value in Health: The Journal of the International Society for Pharmacoeconomics and Outcomes Research,**28*, 769–780. 10.1016/j.jval.2025.01.006 39880195 10.1016/j.jval.2025.01.006

