# Supplementary material for: A direct comparison of the measurement properties of the PROMIS-16 and EQ-5D-5L in the U.S. general population
Source: Qual Life Res. 2026 May 3;35(6):137. doi: 10.1007/s11136-026-04242-8 (PMC13136203; doi:10.1007/s11136-026-04242-8)
Supplement: Supplementary file 1 — Supplementary Material 1 [file 11136_2026_4242_MOESM1_ESM.docx]

**Appendix**

A Direct Comparison of the Measurement Properties of the PROMIS-16 and EQ-5D-5L in the U.S. General Population

Quality of Life Research

Authors:

Minh Pham, Department of Economics, College of Arts and Sciences, University of South Florida, Tampa, Florida, USA

Benjamin M. Craig, PhD, Department of Economics, College of Arts and Sciences, University of South Florida, Tampa, Florida, USA

Tessa Peasgood, PhD, School of Medicine and Population Health, University of Sheffield, UK

Fanni Rencz, DSc, Department of Health Policy, Corvinus University of Budapest, Budapest, Hungary

Corresponding author: Benjamin M. Craig, bcraig@usf.edu; 4202 E Fowler Ave, Tampa, Florida, 33620, USA

**Appendix 1:** Comparison between Respondent Characteristics and the 2021 American Community Survey (ACS)

|  | **Completed, % (n) (n=2577)** | **2021 ACS, %** |
| --- | --- | --- |
| **Age in years** |  |  |
| 18 to 34 | 37 (965) | 29.13 |
| 35 to 54 | 36 (937) | 32.66 |
| 55 and older | 26 (675) | 38.2 |
| **Gender** |  |  |
| Female | 53 (1378) | 50.98 |
| Male | 45 (1167) | 49.02 |
| Other/prefer not to say | 1.2 (32) |  |
| **Race** |  |  |
| White | 75 (1941) | 63.66 |
| Black or African American | 11 (279) | 11.8 |
| American Indian or Alaska Native | 1.0 (25) | 0.89 |
| Asian | 5.5 (143) | 5.97 |
| Native Hawaiian or Other Pacific Islander | 0.1 (3) | 0.14 |
| Some other race | 3.2 (83) | 6.67 |
| Two or more races | 4.0 (103) | 10.87 |
| **Ethnicity** |  |  |
| Hispanic or Latino | 12 (299) | 16.91 |
| Other | 88 (2278) | 83.09 |
| **US regions** |  |  |
| Northeast | 21 (541) | 17.58 |
| Midwest | 22 (556) | 20.67 |
| South | 37 (954) | 38.12 |
| West | 20 (526) | 23.64 |
| **Marital status** |  |  |
| Married | 41 (1056) | 50.41 |
| Widowed | 4 (98) | 5.74 |
| Divorced | 10 (268) | 11.15 |
| Separated | 2 (52) | 1.79 |
| Never married | 43 (1103) | 30.91 |
| don't know/not sure/refuse |  |  |
| **Educational attainment** |  |  |
| 18 to 24 years | 14 (367) | 11.70 |
| 25 years and over | 86 (2210) | 88.30 |
| High school graduate or less | 37 (959) | 32.00 |
| Some college, no degree | 10 (256) | 17.05 |
| Associate's degree | 7 (171) | 7.73 |
| Bachelor's degree | 19 (490) | 18.76 |
| Graduate or professional degree | 13 (334) | 12.16 |
| **Household Income in 2021** |  |  |
| Less than $10,000 | 7 (183) | 6.03 |
| $10,000 to $44,999 | 38 (974) | 30.44 |
| $50,000 to $74,999 | 18 (473) | 16.81 |
| $75,000 to $99,999 | 14 (357) | 12.76 |
| $100,000 to $149,999 | 13 (346) | 16.26 |
| $150,000 or more | 9 (244) | 17.70 |

**Appendix 2a: Response distributions from the item-testing survey and our 2024 survey**

|  | **Item-testing survey (N=5775)** | | | | | **Stand-alone instrument survey (N=2577)** | | | | | | |
| --- | --- | --- | --- | --- | --- | --- | --- | --- | --- | --- | --- | --- |
| **PROMIS-16** | **Without any difficulty** | **With a little difficulty** | **With some difficulty** | **With much difficulty** | **Unable to do** | | **Without any difficulty** | **With a little difficulty** | **With some difficulty** | **With much difficulty** | **Unable to do** |  |
| **Physical function** |  |  |  |  |  | |  |  |  |  |  |  |
| PF1 – “Are you able to go up and down stairs at a normal pace?” | 61  (3524) | 22  (1255) | 12  (712) | 4  (217) | 1  (66) | | 68  (1752) | 20  (508) | 8  (205) | 3  (84) | 1  (28) |  |
| PF2 – “Are you able to go for a walk of at least 15 min?” | 70  (4014) | 16  (946) | 9  (529) | 4  (201) | 1  (79) | | 73  (1878) | 15  (378) | 6  (166) | 4  (97) | 2  (58) |  |
| **Social roles and activities** | **Never** | **Rarely** | **Sometimes** | **Usually** | **Always** | | **Never** | **Rarely** | **Sometimes** | **Usually** | **Always** |  |
| SOC1 – “I have trouble taking care of my regular personal responsibilities” | 42  (2409) | 23  (1326) | 23  (1301) | 9  (515) | 3  (187) | | 62  (1590) | 20  (512) | 14  (372) | 3  (73) | 1  (30) |  |
| SOC2 – “I have trouble doing all of the activities with friends that I want to do” | 45  (2568) | 23  (1330) | 22  (1250) | 8  (445) | 3  (143) | | 55  (1415) | 18  (474) | 17  (447) | 6  (164) | 3  (77) |  |
| **Anxiety (In the past 7 days…)** | **Never** | **Rarely** | **Sometimes** | **Often** | **Always** | | **Never** | **Rarely** | **Sometimes** | **Often** | **Always** |  |
| ANX1 – “I found it hard to focus on anything other than my anxiety” | 43  (2499) | 25  (1444) | 23  (1304) | 8  (430) | 2  (92) | | 45  (1159) | 22  (562) | 23  (580) | 8  (214) | 2  (62) |  |
| ANX2 – “My worries overwhelmed me” | 40  (2294) | 25  (1441) | 23  (1338) | 9  (534) | 3  (164) | | 38  (968) | 22  (561) | 24  (629) | 12  (313) | 4  (106) |  |
| **Depression (In the past 7 days…)** |  |  |  |  |  | |  |  |  |  |  |  |
| DEP1 – “I felt depressed” | 38  (2203) | 23  (1318) | 24  (1371) | 11  (641) | 4  (236) | | 37  (958) | 23  (597) | 24  (622) | 11  (296) | 4  (104) |  |
| DEP2 – “I felt hopeless” | 49  (2805) | 20  (1146) | 20  (1170) | 8  (479) | 3  (167) | | 48  (1236) | 21  (551) | 19  (477) | 9  (229) | 3  (84) |  |
| **Sleep disturbance (See Appendix 3)** |  |  |  |  |  | |  |  |  |  |  |  |
| *Note*. The item-testing survey results are adapted from from *Edelen MO, Zeng C, Hays RD, Rodriguez A, Hanmer J, Baumhauer J, et al. Development of an ultra-short measure of eight domains of health-related quality of life for research and clinical care: the patient-reported outcomes measurement information system PROMIS-16 profile. Qual Life Res. Available from:* <https://doi.org/10.1007/s11136-023-03597-6>*.* Some PROMIS-16’s items (Anxiety, Depression, Sleep disturbance, Pain Interference, Cognitive function, Fatigue) were evaluated in the past 7 days of respondents. The results for sleep disturbance (SLP1 and SLP2) are shown in Appendix 3. | | | | | | | | | | | | |

**Appendix 2b: Response distributions from the item-testing survey and our 2024 survey**

|  | **Item-testing survey (N=5775)** | | | | | **Stand-alone instrument survey (N=2577)** | | | | |
| --- | --- | --- | --- | --- | --- | --- | --- | --- | --- | --- |
| **PROMIS-16** | **Not at all** | **A little bit** | **Somewhat** | **Quite a bit** | **Very much** | **Not at all** | **A little bit** | **Somewhat** | **Quite a bit** | **Very much** |
| **Pain interference (In the past 7 days…)** |  |  |  |  |  |  |  |  |  |  |
| PI1 – “How much did pain interfere with your day-to-day activities?” | 45  (2582) | 28  (1613) | 17  (978) | 8  (429) | 2  (134) | 50  (1292) | 28  (731) | 12  (318) | 7  (174) | 2  (62) |
| PI2 – “How much did pain interfere with your ability to participate in social activities?” | 55  (3134) | 21  (1188) | 15  (836) | 7  (413) | 3  (160) | 64  (1648) | 19  (484) | 9  (227) | 5  (139) | 3  (79) |
| **Cognitive function (In the past 7 days…)** |  |  |  |  |  |  |  |  |  |  |
| COG1 – “I have been able to remember to do things, like take medicine or buy something I need” | 7  (377) | 11  (627) | 18  (1030) | 26  (1460) | 39 (2230) | 25  (637) | 7  (179) | 9  (229) | 16  (424) | 43  (1108) |
| COG2 – “I have been able to think clearly without extra effort” | 4  (251) | 14  (802) | 21  (1182) | 28  (1584) | 33 (1893) | 21  (553) | 9  (244) | 14  (356) | 22  (578) | 33  (846) |
| **Fatigue (In the past 7 days…)** |  |  |  |  |  |  |  |  |  |  |
| FTG1 – “I feel fatigued | 25  (1421) | 35  (2023) | 23  (1339) | 13  (728) | 5  (261) | 29  (736) | 30  (782) | 21  (534) | 12  (307) | 8  (218) |
| FTG2 – “I have trouble starting things because I am tired” | 34  (1977) | 32  (1828) | 20  (1127) | 10  (594) | 4  (241) | 42  (1074) | 25  (646) | 16  (420) | 11  (279) | 6  (158) |
| **EQ-5D-5L** |  |  |  |  |  | **No** | **Slight** | **Moderate** | **Severe** | **Unable** |
| **Mobility (walking)** |  |  |  |  |  | 80  (2058) | 13  (338) | 5  (136) | 1  (34) | 0  (11) |
| **Self-care (washing or dressing myself)** |  |  |  |  |  | 92  (2376) | 6  (149) | 2  (43) | 0  (5) | 0  (4) |
| **Usual Activities (doing my usual activities)** |  |  |  |  |  | 77  (1983) | 16  (405) | 6  (155) | 1  (29) | 0.5  (5) |
|  |  |  |  |  |  | **No** | **Slight** | **Moderate** | **Severe** | **Extreme** |
| **Pain/Discomfort (pain or discomfort)** |  |  |  |  |  | 43  (1099) | 38  (970) | 16  (402) | 3  (86) | 1  (20) |
|  |  |  |  |  |  | **Not** | **Slightly** | **Moderately** | **Severely** | **Extremely** |
| **Anxiety/Depression (anxious or depressed)** |  |  |  |  |  | 45  (1152) | 26  (677) | 18  (474) | 7  (183) | 4  (91) |
| *Note*. The item-testing survey results are adapted from from *Edelen MO, Zeng C, Hays RD, Rodriguez A, Hanmer J, Baumhauer J, et al. Development of an ultra-short measure of eight domains of health-related quality of life for research and clinical care: the patient-reported outcomes measurement information system PROMIS-16 profile. Qual Life Res. Available from:* <https://doi.org/10.1007/s11136-023-03597-6>*.* Some PROMIS-16’s items (Anxiety, Depression, Sleep disturbance, Pain Interference, Cognitive function, Fatigue) were evaluated in the past 7 days of respondents. | | | | | | | | | | |

**Appendix 3: Comparison between the sleep disturbance response distributions of the PROMIS-16 of the item-testing survey and our 2024 survey**

| **Item-testing survey (original publication; N=5775)** | | | | | |
| --- | --- | --- | --- | --- | --- |
| In the past 7 days… | **Never** | **Rarely** | **Sometimes** | **Often** | **Always** |
| SLP1 – “I had problems during the day because of my sleep” | 37 (2141) | 30 (1746) | 21 (1184) | 9 (494) | 3 (183) |
|  | **Not at all** | **A little bit** | **Somewhat** | **Quite a bit** | **Very much** |
| SLP2 – “I had trouble sleeping” | 28 (1593) | 26 (1496) | 28 (1625) | 13 (750) | 5 (285) |
| **Item-testing survey (erratum; N=5775)** | | | | | |
| In the past 7 days… | **Never** | **Rarely** | **Sometimes** | **Often** | **Always** |
| SLP1 – “I had trouble sleeping” | 28 (1593) | 26 (1496) | 28 (1625) | 13 (750) | 5 (285) |
|  | **Not at all** | **A little bit** | **Somewhat** | **Quite a bit** | **Very much** |
| SLP2 – “I had problems during the day because of poor sleep” | 37 (2141) | 30 (1746) | 21 (1184) | 9 (494) | 3 (183) |
| **Stand-alone instrument (N=2577)** | | | | | |
| In the past 7 days… | **Never** | **Rarely** | **Sometimes** | **Often** | **Always** |
| SLP1 – “I had problems during the day because of my sleep” | 38 (977) | 22 (566) | 25 (649) | 10  (266) | 5  (119) |
|  | **Not at all** | **A little bit** | **Somewhat** | **Quite a bit** | **Very much** |
| SLP2 – “I had trouble sleeping” | 28 (720) | 32 (820) | 19 (494) | 13 (341) | 8 (202) |
| *Note:* Between the original publication and the erratum, the two questions on sleep disturbance (SLP1 and SLP2) swapped response option labels and item positions. Additionally, the phrase “because of my sleep” was corrected to “because of poor sleep.” The stand-alone instrument used the phrasing and item order in the original publication, not the erratum, as data collection took place before the erratum was published. | | | | | |

**Appendix 4: Dimension-level and Instrumental-level regression results between EQ VAS and PROMIS-16 and EQ-5D-5L dimensions, constructs. and instruments**

|  | | | | | | **Level 1 to 2** | | | | | **Level 2 to 3** | | | | | **Level 3 to 4** | | | | | **Level 4 to 5** | | | | |
| --- | --- | --- | --- | --- | --- | --- | --- | --- | --- | --- | --- | --- | --- | --- | --- | --- | --- | --- | --- | --- | --- | --- | --- | --- | --- |
|  | **α** | | **p-value** | | **β** | | **p-value** | | **β** | | | **p-value** | | **β** | | | **p-value** | | **β** | | | **p-value** | |  |  |
| **By Dimension** | | | | | |  | |  | |  | | |  | |  | | |  | |  | | |  | |  |
| **PF1** | | 79.88 | | < 0.001 | | -5.71 | | < 0.001 | | -3.97 | | | 0.011 | | -6.65 | | | 0.003 | | 6.61 | | | 0.070 | |  |
| **PF2** | |  | |  | | -6.23 | | < 0.001 | | -4.66 | | | 0.006 | | -4.84 | | | 0.024 | | -3.55 | | | 0.202 | |  |
| **SOC1** | | 81.18 | | < 0.001 | | -1.40 | | 0.165 | | -5.22 | | | < 0.001 | | -0.72 | | | 0.731 | | 0.30 | | | 0.933 | |  |
| **SOC2** | |  | |  | | -6.09 | | < 0.001 | | -5.14 | | | < 0.001 | | -6.76 | | | < 0.001 | | -6.93 | | | 0.003 | |  |
| **ANX1** | | 81.30 | | < 0.001 | | -3.89 | | < 0.001 | | -4.37 | | | < 0.001 | | -2.83 | | | 0.058 | | -7.54 | | | 0.010 | |  |
| **ANX2** | |  | |  | | -1.61 | | 0.130 | | -2.13 | | | 0.051 | | -3.08 | | | 0.019 | | -3.66 | | | 0.120 | |  |
| **DEP1** | | 81.48 | | < 0.001 | | -4.17 | | < 0.001 | | -3.33 | | | 0.003 | | -2.79 | | | 0.045 | | -8.47 | | | < 0.001 | |  |
| **DEP2** | |  | |  | | -0.91 | | 0.404 | | -3.22 | | | 0.006 | | -4.91 | | | 0.001 | | -0.31 | | | 0.907 | |  |
| **SLP1** | | 82.74 | | < 0.001 | | -3.03 | | 0.002 | | -2.16 | | | 0.034 | | -2.72 | | | 0.033 | | -4.02 | | | 0.037 | |  |
| **SLP2** | |  | |  | | -3.00 | | 0.001 | | -3.09 | | | 0.002 | | -3.83 | | | 0.002 | | -6.08 | | | < 0.001 | |  |
| **PI1** | | 81.45 | | < 0.001 | | -5.80 | | < 0.001 | | -5.17 | | | < 0.001 | | -4.50 | | | 0.008 | | -9.50 | | | 0.001 | |  |
| **PI2** | |  | |  | | -3.31 | | 0.001 | | -3.23 | | | 0.031 | | -4.16 | | | 0.028 | | -0.26 | | | 0.925 | |  |
| **COG1** | | 82.13 | | < 0.001 | | -1.64 | | 0.143 | | -0.42 | | | 0.005 | | 0.56 | | | 0.748 | | **9.57** | | | < 0.001 | |  |
| **COG2** | |  | |  | | -4.84 | | < 0.001 | | -3.89 | | | 0.001 | | 0.91 | | | 0.547 | | **7.85** | | | < 0.001 | |  |
| **FTG1** | | 84.36 | | < 0.001 | | -6.51 | | < 0.001 | | -3.04 | | | 0.003 | | -3.28 | | | 0.011 | | -7.58 | | | < 0.001 | |  |
| **FTG2** | |  | |  | | -2.04 | | 0.030 | | -2.99 | | | 0.008 | | -1.36 | | | 0.311 | | -0.54 | | | 0.767 | |  |
| **By Construct** | | | | | |  | |  | |  | | |  | |  | | |  | |  | | |  | |  |
| **ANX1** | | 81.91 | | < 0.001 | | -2.82 | | 0.011 | | -2.82 | | | 0.013 | | -0.68 | | | 0.652 | | -4.39 | | | 0.153 | |  |
| **ANX2** | |  | |  | | -0.12 | | 0.915 | | -0.65 | | | 0.561 | | -0.26 | | | 0.850 | | -1.63 | | | 0.509 | |  |
| **DEP1** | |  | |  | | -2.66 | | 0.022 | | -2.14 | | | 0.060 | | -2.02 | | | 0.156 | | -7.09 | | | 0.004 | |  |
| **DEP2** | |  | |  | | 0.36 | | 0.751 | | -2.41 | | | 0.043 | | -3.98 | | | 0.011 | | 1.87 | | | 0.517 | |  |
| **SLP1** | | 85.21 | | < 0.001 | | 0.04 | | 0.964 | | -0.09 | | | 0.931 | | -0.22 | | | 0.864 | | -2.05 | | | 0.289 | |  |
| **SLP2** | |  | |  | | -1.55 | | 0.097 | | -1.89 | | | 0.057 | | -2.40 | | | 0.044 | | -4.86 | | | 0.001 | |  |
| **FTG1** | |  | |  | | -5.75 | | < 0.001 | | -2.17 | | | 0.033 | | -2.20 | | | 0.089 | | -5.22 | | | 0.002 | |  |
| **FTG2** | |  | |  | | -1.45 | | 0.136 | | -2.25 | | | 0.048 | | -0.82 | | | 0.544 | | 0.23 | | | 0.899 | |  |
| **By Instrument** | | | | | |  | |  | |  | | |  | |  | | |  | |  | | |  | |  |
| **PF1** | | 86.62 | | < 0.001 | | -2.29 | | 0.019 | | -2.97 | | | 0.042 | | -2.67 | | | 0.206 | | 1.50 | | | 0.660 | |  |
| **PF2** | |  | |  | | -2.97 | | 0.005 | | -2.33 | | | 0.143 | | -5.19 | | | 0.009 | | -2.55 | | | 0.333 | |  |
| **SOC1** | |  | |  | | 0.74 | | 0.440 | | -1.95 | | | 0.090 | | -1.66 | | | 0.400 | | -1.20 | | | 0.724 | |  |
| **SOC2** | |  | |  | | -1.75 | | 0.073 | | -0.55 | | | 0.614 | | -1.54 | | | 0.282 | | -2.01 | | | 0.379 | |  |
| **ANX1** | |  | |  | | -0.99 | | 0.317 | | -1.14 | | | 0.263 | | 0.39 | | | 0.772 | | -3.50 | | | 0.210 | |  |
| **ANX2** | |  | |  | | 1.27 | | 0.222 | | -0.65 | | | 0.514 | | 0.15 | | | 0.906 | | 0.63 | | | 0.779 | |  |
| **DEP1** | |  | |  | | -1.21 | | 0.250 | | -0.82 | | | 0.422 | | -0.57 | | | 0.658 | | -4.87 | | | 0.029 | |  |
| **DEP2** | |  | |  | | 0.26 | | 0.800 | | -2.10 | | | 0.049 | | -3.42 | | | 0.014 | | 1.69 | | | 0.517 | |  |
| **SLP1** | |  | |  | | 0.68 | | 0.487 | | 0.31 | | | 0.737 | | 1.55 | | | 0.201 | | 1.86 | | | 0.321 | |  |
| **SLP2** | |  | |  | | -1.07 | | 0.213 | | -0.89 | | | 0.324 | | -0.50 | | | 0.652 | | -3.07 | | | 0.034 | |  |
| **PI1** | |  | |  | | -2.03 | | 0.021 | | -1.69 | | | 0.169 | | -2.45 | | | 0.126 | | -4.55 | | | 0.113 | |  |
| **PI2** | |  | |  | | -0.30 | | 0.762 | | 1.34 | | | 0.344 | | -0.81 | | | 0.649 | | 0.55 | | | 0.838 | |  |
| **COG1** | |  | |  | | -0.04 | | 0.966 | | -0.89 | | | 0.488 | | -2.44 | | | 0.107 | | **3.73** | | | 0.017 | |  |
| **COG2** | |  | |  | | -0.91 | | 0.326 | | -0.07 | | | 0.946 | | 0.06 | | | 0.965 | | -0.27 | | | 0.861 | |  |
| **FTG1** | |  | |  | | -4.14 | | < 0.001 | | -0.82 | | | 0.384 | | -0.76 | | | 0.523 | | -1.68 | | | 0.280 | |  |
| **FTG2** | |  | |  | | 0.90 | | 0.330 | | -0.96 | | | 0.365 | | 1.13 | | | 0.367 | | 0.87 | | | 0.615 | |  |
| **Mobility** | | 85.76 | | < 0.001 | | -4.50 | | < 0.001 | | -4.12 | | | 0.008 | | -4.20 | | | 0.160 | | **14.08** | | | 0.007 | |  |
| **Self-care** | |  | |  | | -2.26 | | 0.098 | | 2.91 | | | 0.258 | | -14.83 | | | 0.031 | | 6.04 | | | 0.557 | |  |
| **Usual Activities** | |  | |  | | -2.76 | | 0.003 | | -6.31 | | | < 0.001 | | -7.22 | | | 0.023 | | 4.84 | | | 0.539 | |  |
| **Pain/Discomfort** | |  | |  | | -4.95 | | < 0.001 | | -4.83 | | | < 0.001 | | -3.37 | | | 0.068 | | -8.14 | | | 0.028 | |  |
| **Anxiety/ Depression** | |  | |  | | -4.79 | | < 0.001 | | -3.13 | | | < 0.001 | | -7.16 | | | < 0.001 | | -5.35 | | | 0.003 | |  |

*Note.* The two items on sleep disturbance (SL1 and SL2) used the phrasing and item order from the original publication, not its erratum (Appendix 3). We regressed the item responses as indicator variables on EQ VAS by dimension, construct, and instrument. For example, the PF regression has eight indicator variables representing four incremental decrements of PF1 and PF2 (row 1). The bolded coefficients are positive and significant (p-value<0.05)

**Appendix 5:** Polychoric’s Correlation Coefficients between PROMIS-16 items

|  | **PF1** | **PF2** | **SOC1** | **SOC2** | **ANX1** | **ANX2** | **DEP1** | **DEP2** | **SLP1** | **SLP2** | **PI1** | **PI2** | **COG1** | **COG2** | **FTG1** | **FTG2** |
| --- | --- | --- | --- | --- | --- | --- | --- | --- | --- | --- | --- | --- | --- | --- | --- | --- |
| **PF1** | - |  |  |  |  |  |  |  |  |  |  |  |  |  |  |  |
| **PF2** | 0.90 | - |  |  |  |  |  |  |  |  |  |  |  |  |  |  |
| **SOC1** | 0.55 | 0.58 | - |  |  |  |  |  |  |  |  |  |  |  |  |  |
| **SOC2** | 0.70 | 0.72 | 0.82 | - |  |  |  |  |  |  |  |  |  |  |  |  |
| **ANX1** | 0.24 | 0.28 | 0.56 | 0.51 | - |  |  |  |  |  |  |  |  |  |  |  |
| **ANX2** | 0.22 | 0.23 | 0.52 | 0.49 | 0.88 | - |  |  |  |  |  |  |  |  |  |  |
| **DEP1** | 0.24 | 0.25 | 0.52 | 0.48 | 0.82 | 0.84 | - |  |  |  |  |  |  |  |  |  |
| **DEP2** | 0.21 | 0.23 | 0.52 | 0.48 | 0.81 | 0.83 | 0.91 | - |  |  |  |  |  |  |  |  |
| **SLP1** | 0.29 | 0.30 | 0.56 | 0.53 | 0.71 | 0.72 | 0.74 | 0.73 | - |  |  |  |  |  |  |  |
| **SLP2** | 0.34 | 0.33 | 0.47 | 0.48 | 0.6 | 0.61 | 0.62 | 0.61 | 0.78 | - |  |  |  |  |  |  |
| **PI1** | 0.68 | 0.69 | 0.59 | 0.66 | 0.47 | 0.44 | 0.45 | 0.43 | 0.52 | 0.54 | - |  |  |  |  |  |
| **PI2** | 0.65 | 0.67 | 0.63 | 0.70 | 0.52 | 0.49 | 0.49 | 0.49 | 0.55 | 0.56 | 0.91 | - |  |  |  |  |
| **COG1** | 0.16 | 0.15 | 0.04 | 0.10 | 0.01 | 0.05 | 0.06 | 0.01 | 0.10 | 0.14 | 0.18 | 0.10 | - |  |  |  |
| **COG2** | 0.12 | 0.10 | 0.03 | 0.04 | 0.08 | 0.05 | 0.04 | 0.10 | 0.00 | 0.08 | 0.10 | 0.03 | 0.88 | - |  |  |
| **FTG1** | 0.41 | 0.41 | 0.56 | 0.56 | 0.64 | 0.65 | 0.67 | 0.64 | 0.73 | 0.68 | 0.59 | 0.59 | 0.19 | 0.08 | - |  |
| **FTG2** | 0.37 | 0.38 | 0.61 | 0.58 | 0.67 | 0.68 | 0.69 | 0.68 | 0.75 | 0.65 | 0.57 | 0.60 | 0.12 | 0.01 | 0.85 | - |
| *Note.* The two items on sleep disturbance (SL1 and SL2) used the phrasing and item order from the original publication, not its erratum (Appendix 3). Cell colors indicate correlation strength: very weak (<0.20, very light blue), weak (0.20–0.39, light blue), moderate (0.40–0.59, blue), strong (0.60–0.79, dark blue), and very strong (≥0.80, very dark blue).  PROMIS-16 dimensions: PF: physical function, SOC: social roles and activities, PI: pain interference, ANX: anxiety, DEP: depression, SLP: sleep disturbance, COG: cognitive function, FTG: fatigue | | | | | | | | | | | | | | | | |

**Appendix 6:** Polychoric’s Correlation Coefficients between EQ-5D-5L items

|  | **Mobility** | **Self-care** | **Usual Activities** | **Pain / Discomfort** | **Anxiety / Depression** |
| --- | --- | --- | --- | --- | --- |
| **Mobility** | - |  |  |  |  |
| **Self-care** | 0.76 | - |  |  |  |
| **Usual Activities** | 0.79 | 0.79 | - |  |  |
| **Pain / Discomfort** | 0.73 | 0.67 | 0.73 | - |  |
| **Anxiety / Depression** | 0.24 | 0.40 | 0.41 | 0.39 | - |
| *Note.* Cell colors indicate correlation strength: very weak (<0.20, very light blue), weak (0.20–0.39, light blue), moderate (0.40–0.59, blue), strong (0.60–0.79, dark blue), and very strong (≥0.80, very dark blue). | | | | | |

**Appendix 7: Differences in ceiling/floor percentages of the PROMIS-16 items**

|  | **Item-testing instrument (Edelen et al.) vs. stand-alone instrument** | | | |
| --- | --- | --- | --- | --- |
| **PROMIS-16**  **items** | **Δ Ceiling, %** | **p-value** | **Δ Floor, %** | **p-value** |
| **PF1** | -7 | < 0.001 | 0 | 0.911 |
| **PF2** | -3 | 0.002 | -1 | 0.005 |
| **SOC1** | -20 | < 0.001 | 2 | <0.001 |
| **SOC2** | -10 | < 0.001 | 0 | 0.183 |
| **ANX1** | -2 | 0.152 | 0 | 0.013 |
| **ANX2** | 2 | 0.065 | 1 | 0.003 |
| **DEP1** | 1 | 0.013 | 0 | 0.952 |
| **DEP2** | 1 | 0.619 | 0 | 0.368 |
| **SLP1** | NA |  | NA |  |
| **SLP2** | NA |  | NA |  |
| **PI1** | -5 | < 0.001 | 0 | 0.815 |
| **PI2** | -9 | < 0.001 | 0 | 0.477 |
| **COG1** | -4 | <0.001 | -18 | <0.001 |
| **COG2** | 0 | 0.980 | -17 | <0.001 |
| **FTG1** | -4 | <0.001 | -3 | <0.001 |
| **FTG2** | -8 | < 0.001 | -2 | <0.001 |
| *Note*. Differences are calculated as stand-alone instrument minus item-testing instrument. Some PROMIS-16’s items and dimensions (Anxiety, Depression, Sleep disturbance, Pain Interference, Cognitive function, Fatigue) were evaluated in the past 7 days of respondents. Cognitive Function items’ ceiling/floor percentages were reversed. The results for sleep disturbance (SLP1 and SLP2) are shown in Appendix 3 due to the erratum published after data collection.    PROMIS-16 dimensions: PF: physical function, SOC: social roles and activities, PI: pain interference, ANX: anxiety, DEP: depression, SLP: sleep disturbance, COG: cognitive function, FTG: fatigue | | | | |
